# Out of pocket expenditure to deliver at public health facilities in India: a cross sectional analysis

**DOI:** 10.1186/s12978-016-0221-1

**Published:** 2016-08-24

**Authors:** Anns Issac, Susmita Chatterjee, Aradhana Srivastava, Sanghita Bhattacharyya

**Affiliations:** Public Health Foundation of India, Plot no. 47, Sector 44 Institutional Area, Gurgaon, 122002 Haryana India

**Keywords:** Out of pocket expenditure, Tips for getting services, Delivery care, Public health facilities, India

## Abstract

**Background:**

To expand access to safe deliveries, some developing countries have initiated demand-side financing schemes promoting institutional delivery. In the context of conditional cash incentive scheme and free maternity care in public health facilities in India, studies have highlighted high out of pocket expenditure (OOPE) of Indian families for delivery and maternity care. In this context the study assesses the components of OOPE that women incurred while accessing maternity care in public health facilities in Uttar Pradesh, India. It also assesses the determinants of OOPE and the level of maternal satisfaction while accessing care from these facilities.

**Method:**

It is a cross-sectional analysis of 558 recently delivered women who have delivered at four public health facilities in Uttar Pradesh, India. All OOPE related information was collected through interviews using structured pre-tested questionnaires. Frequencies, Mann-Whitney test and categorical regression were used for data reduction.

**Results:**

The analysis showed that the median OOPE was INR 700 (US$ 11.48) which varied between INR 680 (US$ 11.15) for normal delivery and INR 970 (US$ 15.9) for complicated cases. Tips for getting services (consisting of gifts and tips for services) with a median value of INR 320 (US$ 5.25) contributed to the major share in OOPE. Women from households with income more than INR 4000 (US$ 65.57) per month, general castes, primi-gravida, complicated delivery and those not accompanied by community health workers incurred higher OOPE. The significant predictors for high OOPE were caste (General Vs. OBC, SC/ST), type of delivery (Complicated Vs. Normal), and presence of ASHA (No Vs. Yes). OOPE while accessing care for delivery was one among the least satisfactory items and 76 % women expressed their dissatisfaction.

**Conclusion:**

Even though services at the public health facilities in India are supposed to be provided free of cost, it is actually not free, and the women in this study paid almost half of their mandated cash incentives to obtain delivery care.

## Plain English summary

Some developing countries have introduced promotional schemes for institutional delivery so that they could improve women’s access to safe delivery care. In India, there is a scheme, titled Janani Shishu Suraksha Karyakram, which entitles women to free delivery care at public health facilities. However, a few studies suggested that the women had to pay for obtaining services even at the public health facilities. To understand the different components of cost of care, the present study conducted a survey of 558 women who had delivered at public health facilities in two districts of Uttar Pradesh.

The majority (97 %) of the women paid from their pocket for services, and the median cost was INR 700 (US$ 11.48); this was half the cash incentive provided by the government scheme. This amount varied between INR 680 (US$ 11.15) for normal delivery and INR 970 (US$ 15.9) for complicated cases. The major component was tips for getting services (consisting of gifts and tips for services) with a median value of INR 320 (US$ 5.25). Women from higher income households (more than INR 4000 (US$ 65.57)/ month), general castes, those who were the first-time mothers, complicated delivery and those not accompanied by community health workers paid more.

The study concludes that the care from the public health facility is in fact not free, and this can discourage women opting for institutional delivery.

## Background

Developing countries suffer from unacceptably high rates of maternal and infant mortality, accounting for 99% of global maternal deaths [1]. One of the primary reasons for this is the lack of access to safe deliveries, especially among the poor, where healthcare access often imposes a considerable financial burden on families [2]. To expand access to safe deliveries and reduce the risk of maternal and newborn emergencies, some developing countries have initiated demand-side financing schemes to promote institutional delivery. Among the South Asian countries, Nepal has the cash incentive scheme and Bangladesh and Pakistan have voucher schemes [3]. Several studies from low and middle-income countries have shown that the cost of care is a major determinant of maternal care utilization and satisfaction with institutional delivery care [4, 5]. Significant associations of cost or affordability of care with maternal satisfaction and the utilization of care in institutional births were found in studies in Nigeria, Zambia, Kenya, Egypt, India, Gambia and Ghana [6–13].

With a high maternal mortality ratio of 178, saving maternal and newborn lives is a key concern in India [14]. The Government of India launched the conditional cash transfer scheme of Janani Suraksha Yojana (JSY) in 2005 to promote institutional deliveries, offering a monetary incentive of INR 1400 (US$ 22.95)^1^ to women delivering in public or accredited private facilities. The program is supported by the Accredited Social Health Activist (ASHA), a community health worker who motivates women to deliver at public facilities and also accompanies them to the facilities [15]. The delivery services, including medicines, tests and food are provided free of cost to encourage women to opt for institutional delivery and offset their related expenditure burden [16]. Later, JSY was modified to Janani Shishu Suraksha Karyakram (JSSK), which included additional services such as support for travel to and from the facility and medical treatment for sick newborn [17, 18].

As a consequence of JSY and JSSK, institutional deliveries in India have increased from 40.7% in 2005-06 to 72.9% in 2009-10 [19, 20]. However, several studies show a persistent and unaccounted high level of out-of-pocket expenditure (OOPE) on maternal care (antenatal, delivery and postnatal), similar to expenditure on other public hospital based services [21–23]. The high OOPE is especially catastrophic for poor households, who are often pushed into further poverty and indebtedness on account of this [24–28]. Though some studies have highlighted OOPE for delivery care, there is less attention to explore the components of OOPE during delivery care at public health facilities.

Our objective was to assess the OOPE incurred by rural Indian women while accessing institutional delivery services at public health facilities and to examine components of OOPE. We also assessed the determinants of OOPE and the level of maternal satisfaction with the OOPE on delivery care. For this study, OOPE refers to all the direct expenditure for delivery care including transportation and services availed from private pharmacies and laboratories due to lack of provision at the public health facility.

## Methods

The present analysis is part of a larger study on women’s experience of care and their levels of satisfaction with maternal services from secondary health care facilities in India. The study followed a mixed-method design with a literature review and qualitative phase preceding the quantitative phase. The literature review aided in exploring the determinants and themes of care from developing countries [4]. This informed the qualitative phase in which perspectives from both the women and healthcare providers were sought. The quantitative phase consisted of a community survey and focused entirely on women’s perspective of delivery care at health facilities. The data presented in this paper is part of the cross-sectional survey.

### Study area

The community survey was conducted during July - August 2014 in two poor performing districts in Uttar Pradesh, one of the high-focus states in India with poor health indicators. The state has high infant mortality rate (50 per 1000 live births) [29], maternal mortality ratio (292 per 100000 live births) and a low percentage of institutional deliveries (45.6 %) [30]. The selected districts had a maternal mortality ratio of 330. The infant mortality rate was 80 and 82 for the two districts whereas the percent of institutional delivery was 35.2 and 42.4 respectively [30]. The survey was aimed at capturing the experience of women with maternal care from secondary level health facilities. Secondary level health facilities are designated to manage complications with childbirth and have provisions for surgical care, blood transfusion and newborn care. These facilities function as the first referral unit for primary level facilities. There were four functional secondary level health facilities in the two study districts.

### Study instrument

The survey instrument comprised of two parts: the first part was a structured interview schedule capturing women’s experience of accessing care from the facility including OOPE on reaching the facility till discharge, and the second part comprised of a scale to assess their satisfaction with care. Based on the literature review and the qualitative study that preceded the survey, items in the instrument were derived from the determinants of structure, process and outcome of care. Among structural determinants, physical environment, cleanliness, availability of human resources, medicines and supplies were included. Determinants of the process of care included interpersonal behavior, privacy, promptness, cognitive care, perceived provider competency and emotional support. Outcome related determinants were the health status of the mother and newborn.

A set of queries focused exclusively on the expenditure across nine categories viz. transportation (hiring vehicle to and from the facility), medicines and supplies (prescribed from the facility), laboratory and diagnostic services, blood transfusion (expenditure for arranging blood), newborn care (in case of neonatal complication, while the mother was still admitted in the facility), expenses on food during her stay in the facility, tips for getting services (in cash or kind: gifts and sweets to facility staff, tips to ambulance driver and facility staff for their services), and other category includes all the expenditure that the women could not classify under the specific categories.

The instrument also included information on socio-economic status and reproductive history of women. In terms of social profile in India, general caste means the non-vulnerable groups and the vulnerable category includes the backward, scheduled and caste and tribe. The maternal satisfaction scale is a 5- point Likert scale with ratings ranging from fully satisfied, somewhat satisfied, neither satisfied nor dissatisfied, somewhat dissatisfied and fully dissatisfied. The scale used had 14 items for understanding satisfaction and included items pertaining to structural, technical and interpersonal aspects of care. The study instrument, prepared originally in English, was translated into the local language (Hindi) of the study area. Translation and back translation was carried out to ensure the exactness of meaning of items in the survey instrument and it was pretested among 20 women in a setting similar to that of the study area.

### Sampling and data collection

The study included women who had delivered at the secondary level health facilities and discharged seven to 42 days prior to the interview. Only women with live births were included in the study. A list of the women with their home address who fulfilled the inclusion criteria was collected from the delivery records of the four secondary facilities in the two study districts. The list contained 2130 women, who had given birth between 20^th^ June and 20^th^ July, 2014. The sample size calculated for the study was 550 with 80% power and 95% confidence interval. The participants were chosen randomly and the randomization sequence was generated using Excel. A sample of 600 women was selected for survey; oversampling was to address dropouts. There were 10 refusals and 32 women could not be traced due to incorrect address. The dropout rate was seven percent. Total 558 women participated in the study. This included both normal and complicated delivery, and there was no stratification on type of delivery during the sampling. The survey was conducted at women’s residences by female researchers who had knowledge of the research topic and local dialect.

### Data analysis

The study refers to OOPE as all the direct expenditure incurred by the women in availing delivery care at the health facility including transportation, all of which are provisioned to be free. This excludes mandatory payments, but includes payment for services from private providers (for transportation, laboratories and pharmacies, etc.) due to shortage or non-functioning of respective services at the public health facility. The costs incurred by relatives or those who accompanied the women were not considered during analysis. Since the women were not aware of all the OOPE related to their deliveries, interviewers sought the help of family members present during the survey to complete the information. Data were collected to calculate direct expenditure only. The total expenditure was calculated by adding all the category-wise expenditure mentioned by the respondents. In order to verify the information, cross questions such as “did you pay from your own pocket for medicine” etc. were included in the instrument. Any discrepancy in the data was noted at the site and clarified from the respondents. Since the respondents were unable to distinguish between mandatory fee and others, information on stipulated fees for services was collected from the respective health facilities.

Survey data were analyzed using IBM SPSS version 19. Data analysis included frequencies, Mann-Whitney test (for comparing the difference between the median expenditure of two groups) and categorical regression (to identify the predictors of OOPE where the dependent variable is on numeric scale while independent variables are on nominal & numeric scales). As the average monthly income and OOPE data were highly skewed, log transformation was done to make the distribution normal.

## Results

All the women paid a registration cost of one rupee at the health facility and this was not included for analysis as it was a mandatory payment specified by the government. Among the 558 women surveyed for the study, total 540 women paid for one or the other type of services from the public health facilities. Hence the present analysis is confined to the sample size of 540.

### Profile of the respondents

The characteristics of the respondents are given in Table 1. The mean age of the women was 25 years. Majority of the women were housewives (98.1 %) and belonged to lower socio-economic strata. For the majority, their husbands were daily wage laborers (43.9 %). Majority women in the sample had a normal vaginal delivery (93.0 %) and were multi-gravida (64.8 %). A significant proportion of them had to travel more than five kilometers to access the health facility (79.8 %). On an average, the women stayed at the facility for two days after delivery.

**Table 1:** Socio-demographic and reproductive profile of respondents (N = 540)

Characteristics		Respondents, n (%)
Age (years)	Mean (SD)	24.9 (4.01)
Education of woman	Illiterate	259 (48.0)
	Literate	281 (52.0)
	Mean years of schooling (SD)	8.4 (3.44)
Religion	Hindu	479 (88.7)
	Muslim	59 (10.9)
	Sikh	2 (0.4)
Caste	General	109 (20.2)
	Other backward caste	232 (43.0)
	Scheduled caste/scheduled tribe	199 (36.9)
Type of household	Nuclear	220 (40.7)
	Joint	320 (59.3)
Occupation of woman	Homemaker	530 (98.1)
	Other work	10 (1.9)
Occupation of husband	Cultivator	149 (27.6)
	Casual labourer	237 (43.9)
	Salaried workers	59 (10.9)
	Self-employed in petty trade/ small scale industry	91 (16.9)
	Unemployed	4 (0.7)
Average monthly household income (INR)^a^	≤4000	299 (55.4)
	>4000	241 (44.6)
Gravidity	1	190 (35.2)
	2 or more	350 (64.8)
Delivery	Normal vaginal	502 (93.0)
	C-section & breech	38 (7.0)
Duration of stay (days)	Mean (SD)	2.1 (1.7)
Distance to health facility	Upto 5 km	109 (20.2)
	More than 5 km	431 (79.8)

^a^Median income is INR 4000

### Out of pocket expenditure (OOPE)

The median OOPE incurred for delivery at the public health facility was INR 700 (US$ 11.48) (Table 2). There was wide variation in the amount paid and it ranged from INR 15 (US$ 0.25) to INR 14400 (US$ 236.07). The women were entitled to receive free transportation to and from the facility; however, in majority of the cases, they had to arrange own transport due to non-responsiveness of government ambulance service. Even among those who availed the service, it was restricted to one side travel. Hence the total expenditure for transportation varied from INR 20 (US$ 0.33) to INR 1634 (US$ 26.79) with a median expenditure of INR 142.5 (US$ 2.34). Expenditure for newborn care, if he/she was referred to a private facility, ranged from INR 100 – 2000 (US$ 1.64 – 32.79) depending on the nature of complication. More than half of the women spent on medicines, cotton pads, syringes and saline, which they bought from private pharmacies. The laboratory facility was not functional round the clock, leading to users relying on private laboratories in the vicinity of the hospital. Tips for getting services, which 86% of women had to incur, included tips to avail government ambulance, and bribes either in cash or kind (for example, distributing sweets) to facility staff for their services. Overcrowding in the facility led to women paying the staff for obtaining a bed in the antenatal and postnatal care wards.

**Table 2:** Item-wise expenditure for delivery care (N = 540)

Items (n)	Median expenditure in INR (min-max)		
	Normal (n = 461)	Complicated (n = 79)	Total (N = 540)
Transportation (*n* = 458)	130 (20-1500)	150 (20- 1634)	142.5 (20 – 1634)
Medicines and supplies (*n* = 298)	100 (15-1200)	110 (35-8000)	100 (15 – 8000)
Laboratory investigations (*n* = 13)	20 (10-200)	450 (100 – 1700)	100 (10 – 1700)
Blood transfusion (*n* = 2)	-	500 (500 – 500)	500 (500 – 500)
Newborn care in private facility (*n* = 3)	^a^	^a^	200 (100 – 2000)
Tips for getting services (*n* = 466)	300 (10-2000)	400 (20-7000)	320 (10 – 7000)
Food (from the facility) (*n* = 15)	200 (20-500)	225 (30-500)	200 (20 – 500)
Any other (*n* = 265)	300 (20-3000)	400 (20-6000)	300 (20 – 6000)
Total expenditure (*n* = 540)	680 (15-5200)	970 (20-14400)	700 (15 – 14400)

^a^Number of cases are not enough to calculate median as they are divided between normal (1) and complicated (2) categories

US$ 1 = 61 INR

The classification of ‘normal’ refers to normal vaginal delivery, and ‘complicated’ indicates all the caesarian and breech deliveries along with maternal complications post-delivery. There was a noticeable difference in the expenditure incurred for normal and complicated cases. Complicated cases had higher OOPE for most of the items compared to normal cases. The difference was striking for laboratory investigations with median INR 450 (US$ 7.38) for complicated cases and only INR 20 (US$ 0.33) for normal cases.

### Determinants of OOPE

Table 3 presents the total OOPE against selected socio-economic and reproductive variables. The significant variables were caste, income, gravidity, type of delivery, and presence of Accredited Social Health Activist (ASHA). The OOPE for other castes was higher than the vulnerable groups of ‘other backward caste, scheduled caste/tribe’ taken together. Similarly, those in the higher income group (above INR 4000 per month) paid more compared to lower income group. The OOPE for multi-gravida was less compared to primi-gravida. The complications associated with delivery (such as C-section and breech cases as well as health complications of mother soon after delivery) incurred higher OOPE than normal delivery. Presence of ASHA during delivery led to less OOPE as the median expenditure was INR 680 compared to INR 980 for the women when ASHA was not present.

**Table 3:** Total expenditure for care across selected variables (*N* = 540)

Variable		Median expenditure in INR (IQR)
Age (Years)	<25	680 (520)
	≥25	740 (665)
Education of woman	Illiterate	670 (510)
	Literate	730 (600)
Religion	Hindu	700 (550)
	Non-Hindu	600 (540)
Caste***	General	850 (780)
	OBC, SC/ST	670 (535)
Income (INR)**	≤4000	620 (520)
	>4000	750 (550)
Gravida**	Primi	785 (678)
	Multi	660 (495)
Sex of baby	Female	655 (493)
	Male	720 (569)
Type of delivery***	Normal	680 (510)
	Complicated	970 (1050)
Presence of ASHA***	Yes	680 (510)
	No	980 (1675)

**p < 0.01, ***p <0.001

Significance level is determined using Mann- Whitney test

US$ 1 = 61 INR

Regression analysis for OOPE provided three significant predictors for OOPE viz. caste, type of delivery and presence of ASHA during delivery (Table 4).

**Table 4:** Categorical regression for OOPE

Variable		Standardized Beta	Standard error
Age (Years)	<25		0.035
	≥25	0.016	
Education	Illiterate		0.025
	Literate	0.011	
Religion	Hindu	0.052	0.034
	Non-Hindu		
Caste*	General	0.098	0.041
	OBC, SC/ST		
Income (INR)^a^		0.025	0.043
Gravida	Primi		0.047
	Multi	0.065	
Sex of baby	Female		0.024
	Male	0.002	
Type of delivery***	Normal		0.060
	Complicated	0.199	
Presence of ASHA*	Yes		0.049
	No	0.103	

*p < 0.05, ***p < 0.001

^a^Natural log of income has taken

### Level of maternal satisfaction with OOPE

Level of satisfaction with delivery care at the secondary level public health facilities was assessed using the Maternal Satisfaction Scale (Table 5). Out of pocket expenditure was one of the least satisfied items marked by the women. Only three percent women were fully satisfied with it. Similarly, the dissatisfaction score was strikingly high with 18.7% which was the second highest among the enquired items.

**Table 5:** Maternal satisfaction with delivery care (*N* = 540)

Item	Level of satisfaction (%)				
	Fully satisfied	Somewhat satisfied	Neither satisfied nor dissatisfied	Somewhat dissatisfied	Fully dissatisfied
Lack of physical abuse	23.0	1.0	1.3	0.4	1.0
Privacy inside labour room	15.9	10.6	4.3	1.0	0.4
Cleanliness of labour room	13.3	12.9	5.1	1.5	1.0
Comfort of bedding	8.9	13.2	8.7	3.7	1.7
Information on progression of labour	8.7	12.1	6.4	5.1	3.8
Waiting time	6.5	7.7	12.6	5.2	4.0
Timely availability of medicine	6.2	9.6	7.6	11.8	2.9
Care and sympathy in labour room	4.2	10.1	14.9	6.2	2.4
Pain relief	3.4	7.4	13.5	9.8	4.0
Out of pocket expenditure	3.0	2.4	2.6	9.0	18.7
	^a^11.9	5.0	7.2	19.1	56.9
Advise on breastfeeding	2.6	4.3	3.8	14.1	13.2
Health advice on cord care	2.0	2.0	2.8	11.3	18.5
Health check-up soon after delivery	1.6	2.4	3.3	10.2	19.1
Opportunity to clarify health concern	0.8	4.2	13.3	10.6	9.2
Total	100	100	100	100	100

^a^Row-wise per cent

## Discussion

The study estimated the OOPE incurred by rural Indian women while accessing institutional delivery services at secondary level public health facilities and also examined its item-wise composition. The study revealed that the services from the government health facility were not free for the women. The OOPE varied from INR 680 to 970 (US$ 11.15 - 15.90) depending on delivery complications. The major component of OOPE was tips for getting services, which comprised of tips for services and gifts in cash or kind. Women from higher income group, general caste, primi-gravida and those without ASHAs paid more compared to their counterparts.

Similar findings have been highlighted in several other studies in which they discussed the high OOPE for institutional delivery in India [28, 31–34]. Recent national level statistics in India revealed that rural population spent on an average INR1587 (US$ 26.0) and urban population INR 2117 (US$ 34.7) to deliver at public facilities [35]. Similar observations were made from other developing countries also. For instance, a study from Bangladesh reported the expenditure for services at government health facilities to be US$ 31.9 for a normal delivery and US$ 117.5 for a caesarean delivery [36]. The median OOPE calculated in the present study was INR 700 (US$ 11.48) with wide variation between normal (INR 680 – US$ 11.15) and complicated cases (INR 970 (US$ 15.90). Based on the data from district level household survey, the estimated mean OOPE for delivery in public health facilities in India is US$ 39 [32]. They found that the OOPE for C-section delivery is six times higher than that for normal vaginal delivery. With the same data, another study showed that median OOPE in public health facilities is INR 1000 (US$ 16.39) and INR 4045 (US$ 66.31) for normal and caesarian deliveries respectively [28]. A study in Uttar Pradesh also found that families pay between INR 500 and 700 (US$ 8.20 – 11.48) to hospital staff at the time of admission or when they have a newborn [22]. The OOPE became catastrophic to families as it surpasses the JSY incentive (INR 1400) in several instances. A study from Orissa highlighted that the JSY incentive accounted to only 25.5% of total maternal expense in rural and 14.3% in urban areas [31].

However, large data sets do not generally allow any detailing to understand the areas where women are paying more. Therefore, the detailed analysis requires in-depth studies utilizing the strengths of mixed methods, like the present study. The major component in OOPE was tips for getting services which include buying gifts to facility staff and tips for services. The median of tips for getting services was INR 320 (US$ 5.25) with a wide range of INR 10 – 7000 (US$ 0.16 – 114.75). The OOPE for complicated cases was higher due to the need for specialized services. In such cases women had to pay for medicines and supplies, laboratory and diagnostic services, blood transfusion and newborn care in private facilities as these services were not fully functional at the public health facilities. A few other studies also reported similar findings and they highlighted how the hospital staff sent family members to nearby private medical shops to buy intramuscular injections of oxytocin and epidosin to speed delivery and also to private diagnostic clinics to conduct ultrasound test [37, 38].

There is difference in OOPE based on socio-demographic characteristics. Those from the general caste and higher income group paid more compared to backward castes and lower income group. In addition to this, the OOPE for primi-gravida was higher than that of multi-gravida. A study from Bulgaria on informal payment in health facilities also found that wealthier, better educated, younger respondents tend to pay more for obtaining better-quality treatment [39]. Studies from India also reported that mean OOPE on delivery was found to be higher among women with higher education and those belonging to a higher wealth quintile, as they have the ability to pay for better quality of service [28–32].

One of the key aspects of promoting institutional delivery, is the role of community health worker- ASHA, in not only motivating the woman to deliver at a health facility but also to accompany her and stay during the entire delivery process [15]. Being familiar with the health system, she is an important link between the community and the health services. The main reason for her to accompany the woman is to inform the woman about the services she is entitled for and also provide emotional support in an unfamiliar environment. In spite of her presence, the study showed that women had incurred OOPE, though it was less when the ASHA was present during delivery care. In some of our qualitative interviews, the ASHAs in fact negotiated with the facility staff in aiding women to avail timely services [40]. There are studies that have highlighted how ASHAs are used by hospital staff to facilitate tips for getting services, and being lower in the health system hierarchy, how they are compelled to negotiate such transaction [22].

The OOPE was among the items rated the least satisfactory on the maternal satisfaction scale. Among the respondents, 76% were dissatisfied (both somewhat dissatisfied and fully dissatisfied) with the payments they made while accessing care at the facility. The remaining 24% included those who were satisfied (both somewhat satisfied and fully satisfied) and also who did not have any particular opinion about the expenditure. The low level of satisfaction with expenditure while accessing healthcare is reported in other studies also [41]. Some studies have reported that attitude to tips for getting services varies from strongly negative to tolerant depending on whether the payment is solicited by the facility staff or offered by the users themselves [39]. Evidence from large-scale population-based surveys in India highlighted the issue of non-utilization of maternal health services due to the cost [42]. As women can evaluate and express their satisfaction with regard to expenditure related to their care, it has implications on utilization of public health facilities for delivery in the future.

### Limitations

The major limitation with the study was the recall bias in reporting the exact expenditure for delivery care at the health facility. In most instances, the male member or the older women who accompanied the pregnant women handled the monetary aspects. Hence, responses from other family members were also recorded. Another difficulty was the inability of some respondents to distinguish between official and unofficial payments. This was clarified with the help of information collected from the respective health facilities on official fees.

## Conclusions

The study highlighted that the services from the public health facilities for delivery care, which were supposed to be free as per safe delivery program of the government, were in fact not free. The women had to incur high OOPE, which varied according to complications associated with the delivery. The item-wise analysis revealed that the major component of OOPE was tips for getting services, followed by expenditure for purchasing medicine, supplies and diagnostic care, which were otherwise mandated to be provided by the health facilities. The findings emphasize the need for a mechanism to curtail tips for services and to improve the availability of medicine and supplies, laboratory and diagnostic services and provision of food from the facility. Though the presence of ASHA helped in reducing the OOPE, they could not eliminate it. There is limited evidence on the areas where women have to incur OOPE within health facilities, which calls for further studies in different geographic locations, to understand the nature and prevalence of OOPE. The very purpose of conditional cash transfer scheme will be lost if a woman has to pay almost half of the monetary incentive in accessing services that are supposed to be free of cost. OOPE on accessing care at public health facilities is one of the key determinants of service utilization, and if not addressed by the health system, can deter women from delivering in public health facilities in future in spite of the cash incentive scheme.

## Abbreviations

ASHA: Accredited social health activist
JSSK: Janani Shishu Suraksha Karyakram
JSY: Janani Suraksha Yojana
OBC: Other backward caste
OOPE: Out of pocket expenditure
SC/ST: Scheduled caste/scheduled tribe

## Footnotes

1 One US$ = 61 INR (during the time of field work -2014)
